# A Clinician-Led, Experience-Based Co-Design Approach for Developing mHealth Services to Support the Patient Self-management of Chronic Conditions: Development Study and Design Case

**DOI:** 10.2196/20650

**Published:** 2021-07-20

**Authors:** Ting Song, Ping Yu, Vida Bliokas, Yasmine Probst, Gregory E Peoples, Siyu Qian, Lauren Houston, Pascal Perez, Mehrdad Amirghasemi, Tingru Cui, Nadeesha Pathiraja Rathnayaka Hitige, Natalie Anne Smith

**Affiliations:** 1 Centre for Digital Transformation, School of Computing and Information Technology Faculty of Engineering and Information Sciences University of Wollongong Wollongong Australia; 2 Illawarra Health and Medical Research Institute University of Wollongong Wollongong Australia; 3 Smart Infrastructure Facility Faculty of Engineering and Information Sciences University of Wollongong Wollongong Australia; 4 School of Psychology Faculty of the Arts, Social Sciences and Humanities University of Wollongong Wollongong Australia; 5 School of Medicine Faculty of Science, Medicine and Health University of Wollongong Wollongong Australia; 6 Illawarra Shoalhaven Local Health District Wollongong Australia; 7 School of Computing and Information Systems Faculty of Engineering and Information Technology University of Melbourne Melbourne Australia; 8 Department of Anaesthesia Wollongong Hospital Wollongong Australia

**Keywords:** mHealth, smartphone, mobile apps, chronic disease, surgery, obesity, theory, community-based participatory research, mobile phone

## Abstract

**Background:**

Despite the increasing use of mobile health (mHealth) services, such as mHealth apps or SMS text messaging services, that support the patient self-management of chronic conditions, many existing mHealth services lack theoretical guidance. In addition, although often the target audience for requirement acquisition at the initial mHealth app design stage, it is a common challenge for them to fully conceptualize their needs for mHealth services that help self-manage chronic conditions.

**Objective:**

This study proposes a novel co-design approach with the initial requirements for mHealth services proposed by clinicians based on their experiences in guiding patients to self-manage chronic conditions. A design case is presented to illustrate our innovative approach to designing an mHealth app that supports the self-management of patients with obesity in their preparation for elective surgery.

**Methods:**

We adopted a clinician-led co-design approach. The co-design approach consisted of the following four cyclic phases: understanding user needs, identifying an applicable underlying theory, integrating the theory into the prototype design, and evaluating and refining the prototype mHealth services with patients. Expert panel discussions, a literature review, intervention mapping, and patient focus group discussions were conducted in these four phases.

**Results:**

In stage 1, the expert panel proposed the following three common user needs: motivational, educational, and supportive needs. In stage 2, the team selected the Social Cognitive Theory to guide the app design. In stage 3, the team designed and developed the key functions of the mHealth app, including automatic push notifications; web-based resources; goal setting and monitoring; and interactive health-related exchanges that encourage physical activity, healthy eating, psychological preparation, and a positive outlook for elective surgery. Push notifications were designed in response to a patient’s risk level, as informed by the person’s response to a baseline health survey. In stage 4, the prototype mHealth app was used to capture further requirements from patients in the two focus group discussions. Focus group participants affirmed the potential benefits of the app and suggested more requirements for the function, presentation, and personalization needs. The app was improved based on these suggestions.

**Conclusions:**

This study reports an innovative co-design approach that was used to leverage the clinical experiences of clinicians to produce the initial prototype app and the approach taken to allow patients to effectively voice their needs and expectations for the mHealth app in a focus group discussion. This approach can be generalized to the design of any mHealth service that aims to support the patient self-management of chronic conditions.

## Introduction

### Background

With the ubiquity of smartphones and the internet, there has been an increasing use of mobile health (mHealth) services in health care systems worldwide to support the patient self-management of chronic conditions, such as hypertension, diabetes, and obesity [1-4]. This has become even more salient since 2020, as the demand for and use of telemedicine services has increased due to the COVID-19 pandemic [4,5]. The use of mobile apps to collect various patient data, such as ecological momentary assessment or experience sampling, has gained momentum to support self-monitoring, develop self-awareness, and promote behavioral change [6,7].

The design techniques of mHealth services have also been continuously evolving, from traditional system design methods focusing on their appearance, functionality, and values to interactive design methods focusing on the way users interact with mHealth services. Common interactive design methods include user-centered design [8-10], activity-centered design [11], and goal-directed design [3,12], focusing on the object, process, and outcome of product use, respectively. To increase user engagement and meet the needs of multidisciplinary collaboration, the development team can also invite all stakeholders, such as domain experts, users, and researchers, to participate in the design and development, known as co-design [13,14]. This is especially relevant when addressing specific diseases or improving physical and mental well-being.

Behavioral change theories and models, such as the Social Cognitive Theory (SCT) [15] and Health Belief Model (HBM) [16], drawn from psychology and sociology [17-19], focus on predicting and explaining human behavior and the wide range of factors that affect these behaviors, such as emotions, habits, and daily routines. These theories provide a roadmap for scientific research and practice [20] and are useful in guiding the implementation of successful mHealth services [21]. mHealth services based on sound behavioral change theories are more likely to lead to positive changes in health behavior [22,23], that is, to lead to successful changes in physical activity and healthy eating [24,25]. Behavioral change techniques include monitoring, intention building, goal setting and planning, progress reflecting, and performance reporting [26,27]. They can be implemented in more interactive and dynamic functionalities in mHealth apps to motivate patients [28].

Despite their increasing popularity, the reported effectiveness of mHealth services for self-management of chronic conditions has been mixed [1,2,29-32]. The frequency with which large numbers of such services enter the market, coupled with the limited time that professional clinicians have available, has inhibited clinician participation in mHealth app design or evaluation [1]. Heterogeneity in mHealth design and purpose also leads to different levels of app usage. For example, apps only being designed as data collection tools instead of comprehensive interventions might not lead to positive changes in health behavior [28]. Similarly, merely providing health information on a regular basis is proven to be ineffective unless reinforcement and motivation are provided [33,34]. As mentioned earlier, inadequate application of the behavioral change theory to guide the design and implementation of mHealth services also leads to unsatisfactory intervention effects [23,31,35,36]. In addition, although often the target audience for requirement acquisition at the initial mHealth app design stage, it is a common challenge for patients to fully conceptualize their needs for mHealth services that support them to self-manage chronic conditions [32]. These may lead to the ineffectiveness of mHealth services [3,9,10,13,14].

### Research Aim

To address the abovementioned limitations for the design of effective mHealth services that support the patient self-management of chronic conditions, this study proposes a novel approach with the initial requirements for mHealth services proposed by clinicians based on their experiences in guiding patients to self-manage chronic conditions. We adopted a co-design approach with multidisciplinary collaboration to improve knowledge about patients’ need for mHealth services.

### A Design Case of mHealth Services for Preoperative Obesity Management

Obesity has increasingly become a global public health challenge, with 5.9 million Australians (31.3%) having a BMI ≥30 kg/m^2^ in 2017-2018 [37]. Obesity can complicate procedures such as siting intravenous cannulae and inserting endotracheal tubes. It may affect weight-based decisions such as ventilator settings or drug doses and can also make surgical access more difficult [38,39]. Obesity is also a risk factor for short-term postoperative complications such as infection, deep vein thrombosis, poor wound healing, blood loss, respiratory problems, and myocardial infarction [38,40,41]. In Australia, the average waiting time for elective surgery in public hospitals is 41 days [42]. Losing weight and improving preoperative fitness through lifestyle changes during this period, known as prehabilitation, is gaining increasing attention [43,44].

Common methods of prehabilitation include engagement in regular physical activities, dietary optimization, and psychological support [45,46]. Current evidence suggests that higher preoperative fitness can lead to fewer last-minute cancelations, better postoperative outcomes, and shorter patient waiting times [47-49]. However, many local health care systems do not have enough resources to help patients with obesity improve their preoperative fitness for surgery, even when the period between the booking and performance of surgery can be up to 12 months. Moreover, dietary modifications and changes in physical activity are difficult to maintain [50,51]. Therefore, innovative methods are needed to encourage and motivate patients with obesity to improve their physical fitness, dietary habits, and mental well-being before elective surgery.

To date, the use of mHealth services to improve preoperative fitness is in its infancy [52,53]. There are bariatric surgery-specific apps available in app stores; however, no studies have reported the use of a behavioral theory to guide the development of mHealth services to deliver health and weight management coaching to patients with obesity before elective surgery [54,55]. Considering that there is still room for further research on embedding a relevant behavioral theory within mHealth apps to improve effectiveness, a case is presented to illustrate our innovative approach to designing an mHealth app that supports self-management of patients with obesity in their preparation for elective surgery.

## Methods

### Overview

**Figure 1:**
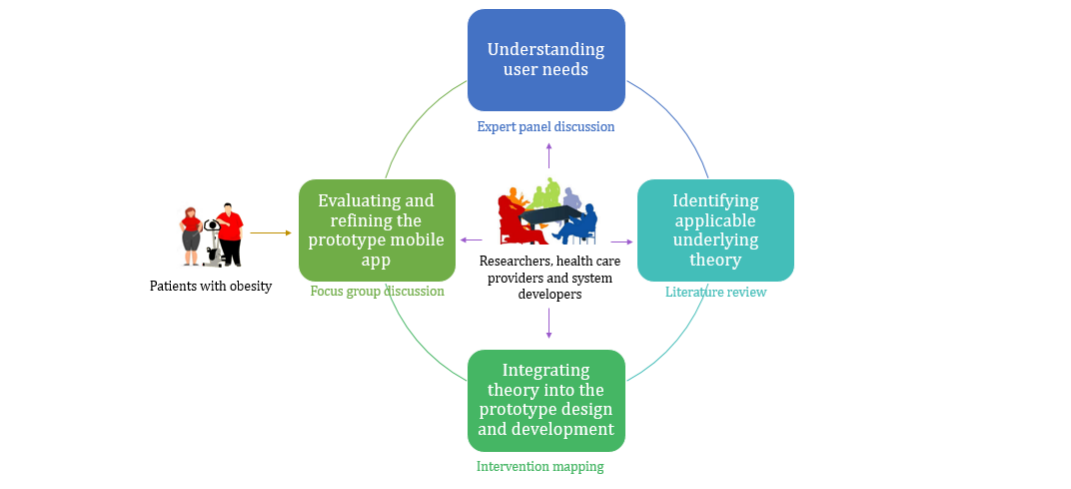
Stakeholders, procedures, and methods of the intervention development process.

A clinician lead co-design approach was undertaken by a multidisciplinary team for designing mHealth services to support the patient self-management of chronic conditions. The experience-based co-design principle [56] and the guidelines for developing complex interventions to improve health and health care published by the UK Medical Research Council [57] have been followed to design the mHealth app. Experience-based co-design claims that all stakeholders, including researchers, developers, and service users, participate in the design process and develop a set of feasible service plans or care paths by gathering their experiences [56,58,59]. The Medical Research Council framework defines a series of actions for intervention development [57]. On the basis of these references, we formulated four iterative phases for the prototype mHealth app design: understanding user needs, identifying applicable underlying theories, integrating theory into the prototype design and development, and evaluating and refining the prototype mobile app (Figure 1 [57]).

In phase 1, we aimed to understand user needs through an expert panel discussion. The panel included domain experts in the medical field and health information systems. Domain (medical) experts can put forward specific challenges that patients may face in the self-management of chronic diseases based on their clinical experience and can also provide various targeted professional assessments and interventions for integration into the mHealth service. Health information system experts can effectively transform this information into system requirements and design considerations to achieve an optimal design solution. In phase 2, a literature review was conducted to compare the relevant theories and to select the optimal one, as suggested by the empirical studies to guide the mHealth service design, in accordance with the guidance of Nash and Barnier [20] and Yang and Van Stee [21]. In phase 3, the selected theory was implemented in the prototype mHealth design and development using the intervention mapping approach, that is, using theory and the corresponding constructs to propose the relevant functions of the mHealth service, which is widely used for the development of theory-based health promotion programs [60]. In phase 4, a focus group discussion was conducted with patients. The patients were provided with the opportunity to interact with a living prototype mHealth service, which facilitates the identification of their needs and their desired functions of the mHealth service. The tangible feedback provided the research team with evidence to further improve the design of mHealth services.

### Phase 1: Understanding User Needs

A multidisciplinary team of 12 experts conducted panel discussions at the University of Wollongong, Australia, to understand potential user needs; 8 team members hold a PhD and 4 hold master’s or bachelor’s (with Honors) degrees as their highest qualification. In total, 9 panel members had more than 5 years of research experience related to health sciences.

A clinical anesthetist (NAS), with a 20-year clinical experience in Wollongong Hospital, described the issues related to obesity, anesthesia, and surgery, which provided information on user needs from a clinical perspective. She noted that the challenge of obesity is particularly problematic in the local area, the Illawarra Shoalhaven Local Health District, New South Wales, Australia, in which the prevalence of obesity is higher than the national figure (36% compared with 28%) [61]. According to local audit data, one-third of patients scheduled for elective surgery waited more than three months between booking and operation, and about half of them gained weight while on the waiting list. In Wollongong Hospital, approximately 55% of the 6000 patients who undergo elective surgery each year have obesity [62].

Together with an eHealth researcher (PY), these authors proposed that an mHealth intervention could potentially address the needs of this population. An accredited clinical psychologist (VB), accredited practicing dietitian (YP), and accredited exercise physiologist (GEP) provided specific input in their areas of expertise. Researchers in health information systems and software engineering were involved in designing technical solutions for delivering the intervention.

### Phase 2: Identifying Applicable Underlying Theory

After understanding the preliminary user needs, the researchers conducted a literature review to identify applicable underlying theories to guide the app design. Three interdisciplinary databases (Scopus, PubMed, and PMC) were searched, which allowed the inclusion of peer-reviewed English-language journal articles. Terms, that is, Medical Subject Headings and their variants, applied were *theory or model*, *intervention or program*, and *behavioral change*. Empirical studies and systematic reviews that reported the explicit use of theory to guide lifestyle-related behavioral changes were included in the study. Studies that did not report why or how the theory was used were excluded. The data were extracted in a Microsoft Excel spreadsheet for constant comparison and analysis. The team then selected the most suitable theory to guide the prototype design for this study.

### Phase 3: Integrating Theory Into the Prototype Design and Development

After selecting the SCT as the most applicable theory, we used it as a framework to integrate the prototype design, following the intervention mapping protocol [60]. First, we listed all user needs based on their understanding of health issues, risk groups, behavioral and environmental determinants, and available resources. Second, we listed the constructs of the selected theory and the evidence-based intervention techniques used to guide the behavioral changes that fit the abovementioned intervention context. Third, we mapped these intervention techniques to the app design to build different functional modules. Fourth, we designed the system architecture, functional modules, user interface, and database and integrated these components into a coherent program (ie, that of a prototype mobile app), through several iterations. React Native, an open-source framework that uses JavaScript and React to develop native, iOS, and Android mobile apps, was used in the development [63].

### Phase 4: Evaluating and Refining the Prototype Mobile App

The prototype mobile app was pilot used by patients with obesity to gauge their perceptions of its usefulness and usability. The pilot trial was conducted in November 2019 in two focus group discussions with 6 people per group. Participants were recruited via purposive sampling of patients with obesity who were undergoing weight loss treatment at a hospital in the South-Western Sydney Local Health Service via existing networks. The inclusion criteria were patients who (1) were aged ≥18 years, (2) had a BMI ≥35 kg/m^2^, (3) were English-speaking with self-elected adequate reading skills, and (4) provided informed consent to participate in the study. A clinic nurse initially discussed the project and sought verbal consent from each patient to participate in the focus group discussion. One week later, patients were sent a text message asking for confirmation of their verbal consent. Ethical approval for the study was obtained from the University of Wollongong and Illawarra and Shoalhaven Local Health District Health and Medical Human Research Ethics Committee (2018/175; HREC/18/WGONG/64).

One researcher (LH), with prior training in research theory and experience in conducting and observing qualitative research, moderated the semistructured focus group discussions. For each group, she gave a brief introduction to the participants and asked them to sign a written consent form. She then distributed 3 mobile phones and 3 mobile tablets with the app preinstalled for all participants and instructed them on its use. After the time spent with the app, a semistructured list of questions was deliberated in the group, discussing the relevant functions of the app (Multimedia Appendix 1). The conversation continued until all of the relevant issues and opinions were openly raised and discussed, beyond answering the interview questions.

The focus group discussions were audio-recorded and transcribed verbatim; 3 other researchers were present as observers and took notes to record the nonverbal characteristics of the focus groups, such as gender. The total discussion time for each group was approximately 1 hour and 30 minutes.

The transcripts were analyzed using a content analysis approach to capture patient feedback [64]. Each original sentence was judged by 2 researchers independently to see if it contained a suggestion that would be useful for improving app functionality. These suggestions were listed in point form and circulated to the expert team. The developers discussed the approved and feasible suggestions for further modification.

## Results

### Phase 1: Understanding User Needs

The expert panel discussion proposed three kinds of potential needs for patients with obesity to improve fitness before surgery: motivational needs, educational needs, and supportive needs.

#### Motivational Needs

Many individuals find it difficult to maintain sufficient motivation to lose weight over time. Many have repeated failed experiences, often with initial weight loss followed by regaining weight, which can further decrease confidence and motivation [50,65]. It is well recognized that supporting motivation is essential for sustainable behavioral change [3,27,66,67].

#### Educational Needs

Educating patients with obesity about the general health risks associated with obesity remains important. Most studies have not considered risks that are specifically associated with anesthesia and surgery, and many do not realize that obesity itself poses an additional perioperative risk. Therefore, we felt it important to educate patients faced with upcoming surgery about the related risks in a manner that was personally tailored to their specific situation. The aim of increasing awareness was to capitalize on the potential *teachable moment* of surgical booking to encourage behavioral change [68-70].

#### Supportive Needs

Even if patients are aware of obesity-related health risks, many need ongoing guidance about strategies and encouragement to stay motivated in changing their lifestyles to lose weight. Therefore, it is essential to provide timely, relevant information and regular interactions to improve patient skills and encourage a positive attitude toward behavioral change. Regular reminders are an effective means of providing personalized, targeted support [66,67].

### Phase 2: Identifying Applicable Underlying Theory

In addition to the SCT and HBM, theories relating to health behavior include the Theory of Planned Behavior [71], Self-determination Theory [72], Transtheoretical Model [73], Community Organization Model [74], and Diffusion of Innovation Theory [75] (see the detailed comparison in Multimedia Appendix 2).

As the HBM and Theory of Planned Behavior focus only on rational reasoning, ruling out unconscious, spontaneous behavior and its emotional effects [53,76], and the Self-determination Theory is confined to explaining only behavioral motivations, none of these was considered suitable as a guide for the development of interventions that provide comprehensive health care support, such as health education and reminders [72]. The Transtheoretical Model cannot explain how an individual thinks he or she is ready to cope with a change or not, which would have caused difficulty in mapping intervention techniques to the behavioral determinant factors, thus weakening the explanatory capacity of the theory [77]. The Community Organization Model and Diffusion of Innovation Theory are both focused on initiatives to support community health promotion at the population level; therefore, they are not intended to guide the development of interventions to support behavioral change for individual patients [78]. Finally, the SCT was selected to guide the design and development of the app.

The SCT explains an individual’s behavior through a reciprocal model of interactions among behavior, personal factors, and the social environment. It is a theory that synthesizes a wide range of behavioral, cognitive, and environmental determinants of behavioral change, such as self-efficacy, observational learning, outcome expectations, and additional reinforcement [46]. This theory not only explains the behavior of individuals under rational circumstances but also describes the influence and interaction of internal cognitive and external environmental influences on human behavior [15], so it can be applied to guide the design of complex interventions to support the management of chronic conditions. Moreover, the SCT considers that people learn not only through their own experience but also through imitating behaviors and the results of these behaviors. We felt that this was consistent with the purpose of this study to provide professional coaching to guide patients with obesity in terms of preoperative weight loss and fitness improvement. Therefore, the SCT was chosen as the most appropriate theory to guide the intervention design in our study.

### Phase 3: Integrating Theory Into the Prototype Design and Development

The SCT contains seven significant constructs: self-control, self-efficacy, expectations, expectancies, reinforcement, behavior capacity, and observational learning. All of these constructs were used to guide the design of conceptual intervention techniques and functions in the app (Table 1).

**Figure 2:**
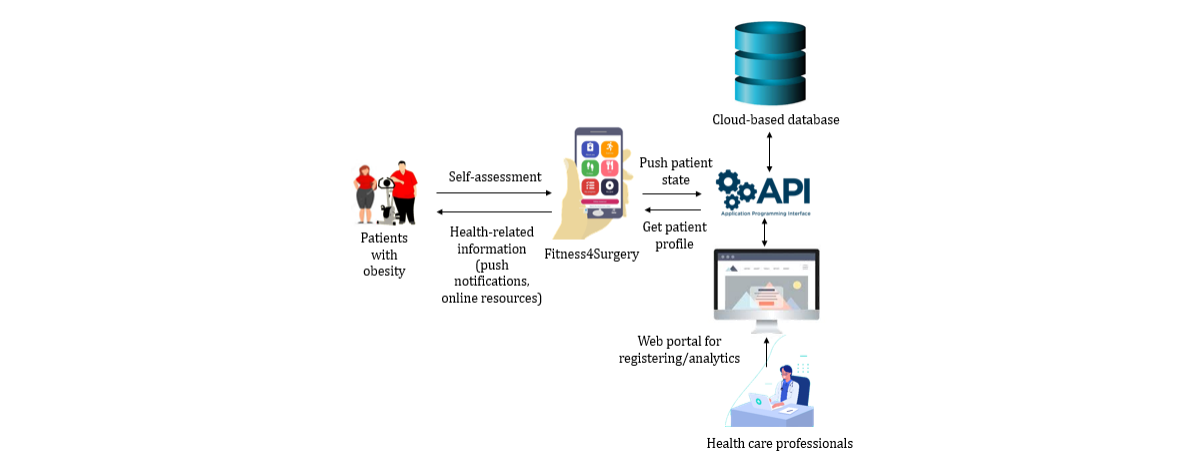
Working mechanisms of Fitness4Surgery.

Our app, Fitness4Surgery, consists of two interfaces (Figure 2 and Multimedia Appendix 3): a mobile interface for patients to self-manage their obesity and a web-based portal for the health care administrators to edit, modify, and update the content of push notifications, view patients’ interactions with the mobile app, and formulate text interventions.

At the initial log-in to the app, patients are requested to answer the questionnaire surveys about their mobile phone usage experience, level of physical activity, diet, psychological well-being, and preoperative health. The system classifies their level of function as *high* or *low* based on these data and will provide corresponding push notifications automatically; 96 push notifications were designed by four domain experts, that is, the clinician, the clinical psychologist, the practicing dietitian, and the exercise physiologist, based on advice from the Australian Dietary Guidelines and Australia’s Physical Activity and Sedentary Behavior Guidelines and the research evidence [79-81] (see exemplar notifications in Table 2).

**Table 1:** User needs, theoretical construct, conceptual intervention techniques, and app functionalities.

User needs and theoretical construct		Conceptual intervention techniques	App functionalities
**Motivational needs**			
	Self-control	Let users set goals and encourage them to monitor their own behavior toward the achievement of these goals	Build a functional module named “My Goals”
	Self-efficacy	Let users start by setting small, progressive, and realistic goals	Provide some simple exemplar goals in “My Goals”
	Expectations	Let users know about the benefits of fitness for surgery	Provide an introductory video about the significance of fitness for surgery
	Expectancies	Allow users to track and monitor their own changes in weight, diet, and physical activity. Provide feedback and let users evaluate what they value	Build a functional module named “My Steps” to record the number of steps each day Build a functional module named “Surveys” and ask the users to complete these surveys Send different push notifications according to different responses for feedback
	Reinforcements	Let users recognize and praise their achievements by specifying rewards	Make a trophy pop out once a user achieves their goal
**Educational needs**			
	Behavior capacity	Teach users how to self-manage diet, physical activity, mood, and medical conditions	Translate relevant educational information into push notifications and send them to the users
	Observational learning	Let users watch some actions and outcomes of others’ behavior	Provide an introductory video about how to achieve fitness for surgery
**Supportive needs**			
	Reinforcements	Remind users to perform the behavioral change toward fitness for surgery Provide users with toolkits and resources that make the new behaviors easier to perform	Send push notifications Build a functional module named ”My Resources“

**Table 2:** Exemplar push notifications in the four domains.

Domain and content			Risks for the group of patients
**Physical activity**			
	"Limit sitting time to a maximum of 30 minutes."	Low	
	"Limit sitting time to a maximum of 20 minutes."	High	
**Diet**			
	"Each day this week take a photo of your meal."	High or low	
**Psychology**			
	"You’ve got this! Stick with it. Set goals and take them one step at a time. It will be worth it!"	High or low	
**Medical advice**			
	"Reducing weight before your surgery can benefit your recovery and prevent unwanted complications. Work out your target weight by speaking to your general practitioner or head to Get Healthy NSW^a^ by phone or on the web for more help."	High or low	

^a^NSW: New South Wales.

Patients with obesity are asked to set goals on the app. Once a goal is reached, a trophy pop will be displayed on the screen as a reward. They will be presented with links to existing health resources, such as the Heart Foundation [82] and Get Healthy New South Wales [83]. The patient-only use of the app keeps the data entered by the patient confidential. Patients can show the records to their doctors for discussion if they wish. There is a large variation in each clinician’s approach to managing obesity [84]; therefore, the app did not cover this function. A notification will be automatically sent to patients every month to ask them to update their responses to the surveys.

### Phase 4: Evaluating and Refining the Prototype Mobile App

#### Evaluation

##### Overview

The focus group participants reported two perceived benefits of the app: usefulness and ease of use. They also discussed areas that needed improvement for the four functional modules, that is, Survey, My Goals, My Resources, and push notifications (Table 3).

**Table 3:** Data analysis of the focus group discussion.

Theme, category, and topic				Exemplar quote
**Perceived benefits**				
	**Usefulness**			
		Evaluate health conditions and health literacy before surgery	“Well, yes it’s a good app, because it’s got a lot of benefits; our progress, our goals, resources we can use, so, yes, it’s probably good for everybody here.” [group 1-05, female]	
		Access to health-related knowledge, skills, and referrals	“Looking at the resources that they’ve offered, it’s a personal lifestyle app.” [group 2-05, male]	
		Improving the patients’ motivation	“If it’s just going to be a tool for yourself, perhaps an inspirational tool?” [group 1-01, male]	
	**Ease of use**			
		Easy to use the system	“Yes, I think it’s good, it’s quite easy to follow, because I’m not very good at this, but I found it quite easy.” [group 1-02, female]	
**Improvement**				
	**Survey**			
		Inflexibility	“I physically cannot walk ten minutes, so my answer to the question is zero, but when I tried to complete the page, it’s telling me it’s incomplete, so all the information I put in is going to be junked (deleted).” [group 2-03, female]	
		Ambiguity	“Click on what? No, something’s wrong.” [group 1-02, female]	
	**My Goals**			
		Insufficient feature	“When you complete the survey at the start about you, yes, you’ve got a baseline, but over a period of time, the app is not collecting anything.” [group 2-02, male]	
		Suboptimal interface design	“What about a graph about filling out or achieving the goals and seeing...something visual.” [group 1-05, female]	
		Lack of tracking	“...is there a place where you can monitor your weight loss as you go?” [group 1-06, female]	
	**My Resources**			
		Insufficient information and referrals	“...it does need more resources in it” [group 2-02, male]	
	**Push notifications**			
		Personalization	“Maybe there’s an option that you can turn that setting on or off...So, I like to stipulate what time (messages arrive).” [group 1-06, female]	

##### Perceived Benefits

Overall, the participants reported that the app could be useful, and they were looking forward to using this product for their surgical preparation. They felt that the app would help them (1) evaluate their health conditions and health literacy before surgery; (2) access health-related knowledge, skills, and referrals; and (3) improve their motivation by setting goals and rewarding their achievements. Some praised the ease of use of the app, even if they were not proficient in using smartphones.

##### Improvement

###### Survey

The participants raised two issues regarding the module. First, the operability of the system was not sufficiently flexible. They found that some questions in the questionnaire were irrelevant to their own situation. As all questions were mandatory, it was difficult to continue entering the data. Therefore, they suggested that some questions should be optional, which would allow them to skip the questions that were irrelevant to them or move through this section more quickly if they wished to. The second was semantic ambiguity. A few participants were confused about the meaning of certain questions and did not know how they should answer. They suggested modifying the expression of some questions to make them easier to understand.

###### My Goals

The participants raised three issues regarding the module. The first issue was monotony. They stated that it was boring because of its simplistic function and presentation, with an inadequate interface design. They suggested having some preset common goals as examples for users to choose from while retaining the flexibility to set their own goals. In particular, they felt that the color scheme was uninspired, with insufficient incentives provided to achieve their preset goals. They strongly requested the use of different colors, shapes, and icons to enrich the interface, with the provision of visual rewards once goals were completed, such as the appearance of animated trophies or fireworks. The second issue was functional. The participants felt that the timeframe for goals was important but not currently well defined. The third issue was the lack of a tracking mechanism. They felt disappointed because they could not monitor their progress toward achieving their goals. They also described several functions that they expected or found in other apps.

###### My Resources

The participants were generally satisfied with this module, except that some requested the provision of more psychological support. One participant requested a recipe section in the app.

###### Push Notifications

The main focus of this discussion was on the optimal delivery time and frequency of these notifications. Some felt that daily notifications would help remind them of healthy routines, whereas others felt that excessive reminders could be overwhelming. One suggestion that was supported by the participants in both groups was that the app should allow users to set the timing that suited them to receive push notifications and that they could turn the reminder on or off themselves.

##### After Focus Group Refinements

**Figure 3:**
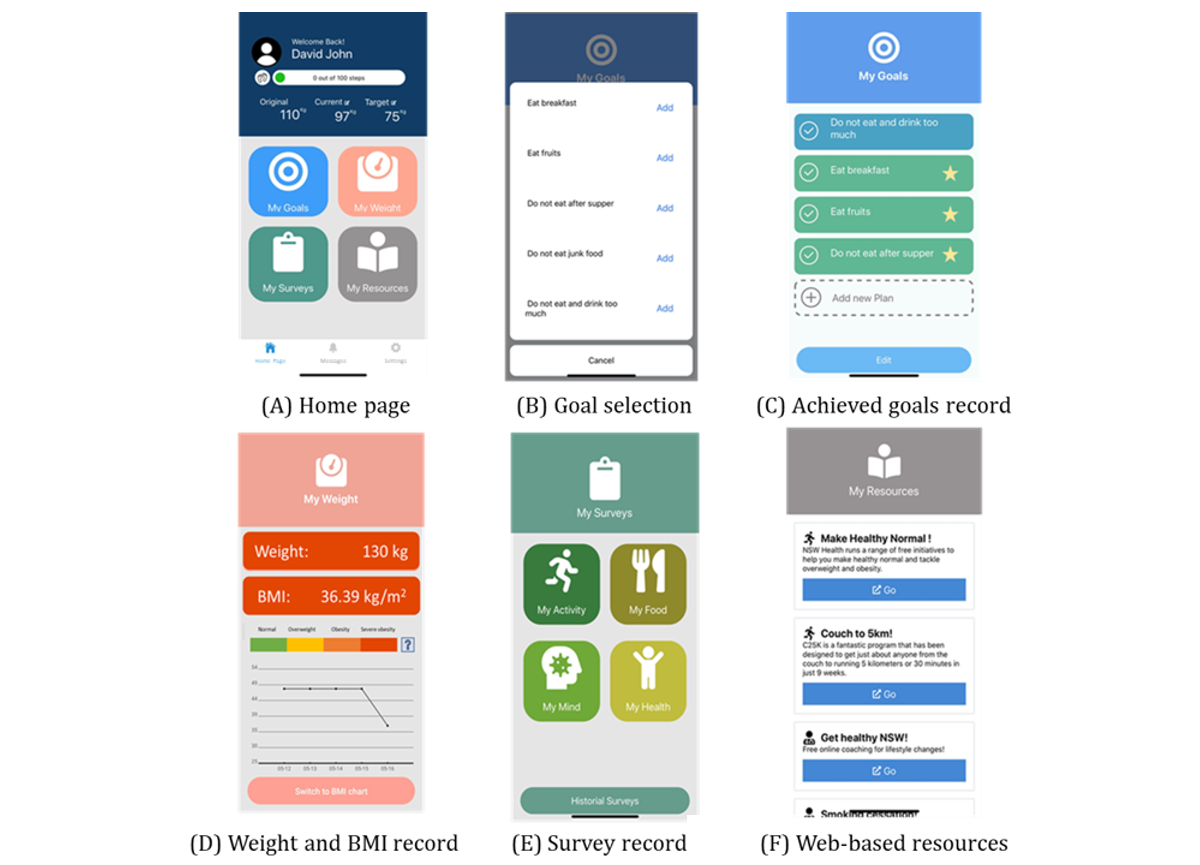
Screenshots of key functional modules of the refined Home page of the Fitness4Surgery app. (A) Home page, (B) goal selection, (C) achieved goals record, (D) weight and BMI record, (E) survey record, and (F) web-based resources.

The refined design of the interface was simplified into three pages: *Home*, *Messages*, and *Settings* (Figure 3). The Home page consists of two major parts: user information and four buttons—My Goals, My Weight, My Surveys, and My Resources. The user information includes the name, profile, step count, and weight record. The display of weight at multiple points over time encourages users to progress toward their target weight.

In the My Goals module, the ability to select preset goals from a list was added to the free-text space. Once a goal is achieved, the user clicks once to record it and receives a star as a reward; 10 stars can be exchanged for a trophy and 10 trophies for a firework. A progress tracking function was also incorporated into the module.

A new module, My Weight, was added to the Home page, where users can read the changes in weight and BMI, both numerically and graphically. This allows users to track and monitor their own changes in weight, which addresses the application of expectancies in the SCT.

Apart from color and layout adjustments, the functions of My Surveys and My Resources remained unchanged. Users were able to answer the surveys at any time, and the results were recorded in a new area accessible for later review.

## Discussion

### Principal Findings

To date, limited evidence exists regarding the use of a behavioral theory to guide the development of mobile services to support the patient self-management of chronic conditions, particularly in the context of prehabilitation for patients with obesity who are awaiting surgery. Guided by an existing framework for developing complex interventions to improve health and health care, this multidisciplinary study proposed a clinician-led, experience-based co-design approach and implemented it in developing a prototype mobile app, Fitness4Surgery, to provide guidance and support for patients with obesity to change lifestyle, lose weight, and improve fitness. The approach consisted of four iterative phases: understanding user needs, identifying theory, integrating theory into the design, and evaluating the prototype. In each phase, we engaged as many relevant stakeholders as possible for the consultation and gathered multiple sources of evidence from expert panel discussions, literature review, intervention mapping, and focus group discussions. Therefore, we adopted an evidence-based approach to design our mHealth service by drawing on experiences from clinicians, patients, researchers, and software developers.

To the best of our knowledge, this study is the first to articulate a detailed *co-design* approach that leverages the clinical experiences of clinicians and multidisciplinary teams to produce the initial prototype app. The prototype allowed the patients who participated in the focus group discussions to directly interact with the mobile app and experience its functions. This *hands-on* experience enabled them to draw on their needs and expectations for the mHealth app. The research output is useful for designing innovative digital interventions to provide just-in-time support for patients, which is low cost and easy to access [85]. This provides a useful alternative solution to address the service gap due to a shortage of funding and lack of human resources to provide these services face-to-face to vulnerable patients in the public health care system. mHealth services are also advantageous in the current period of the COVID-19 pandemic when social distancing is required [4,5]. Compared with similar studies published, our research contributes three distinct innovations to advance the design of mHealth apps (Table 4).

**Table 4:** A comparison of the contribution of this study and the existing literature.

Study	Aim	Design technique	Theory used	Theory mapping	User or clinician design and test
This study	To support patients with obesity to lose weight and improve fitness before surgery	Experience-based co-design	SCT^a^	Yes	Yes, expert panel discussion and focus groups
Smaradottir et al, 2020 [10]	To support chronic pain management	User-centered design	No	N/A^b^	Yes, a cocreation workshop
Wachtler et al, 2018 [8]	To improve treatment allocation for depression	User-centered design	Theory of agent-oriented modeling	No	Yes, two focus groups
Morita et al, 2019 [9]	To support asthma self-management	User-centered design	No	N/A	Yes, semistructured interviews
Duan et al, 2020 [3]	To improve patient compliance with hypertension self-management	Goal-directed design	HBM^c^ and technology acceptance model	No	Yes, persona establishment (questionnaire and interview)
Fore et al, 2013 [12]	To support chronic care for pediatric inflammatory bowel disease	Goal-directed design	No	N/A	Yes, semistructured interview
Woods et al, 2019 [13]	To support heart failure self-management	Nurse-led co-design	No	N/A	Yes, interviews and workshops
Martin et al, 2020 [14]	To improve obesity-related health behaviors of adolescents	Co-design	Behavior change wheel, positive psychology, SDT^d^, and nudging theory	No	Yes, workshop

^a^SCT: Social Cognitive Theory.

^b^N/A: not applicable.

^c^HBM: Health Belief Model.

^d^SDT: Self-determination Theory.

First, only a few of the mHealth developments for supporting behavioral change in recent years have reported the use of a specific theory [3,8,14]. Our research analyzed and compared common theories related to behavioral changes. In addition to guiding ideology at a high level, the theory-based design also involves the in-depth mining and analysis of all relevant constructs in theory and the mapping of the constructs to each user’s needs to conceptualize a series of corresponding intervention techniques. These techniques were then converted to different real functionalities and were built into different function modules in the app, ensuring scientific rigor and practicality. The three proposed types of user needs are consistent with those addressed by similar mHealth interventions in alcohol and HIV areas [66,86]. Huygens et al [87] conducted a comprehensive qualitative focus group discussion with patients with chronic diseases to explore their expectations and needs for using mHealth for self-management purposes. Patients with obesity perceived need fulfillment and disease control as determinants of their willingness to use the app, which reflects the advantages of ecological momentary assessment and intervention [6,87,88].

Second, user interviews are traditionally conducted to gather user needs from the first stage [3,8-10,12-14], but in our study, clinicians proposed patient needs as the first step toward app content and design. This approach had two advantages. The first advantage was that clinicians’ suggestions based on scientific and public health reports and their years of field observation were practical and valuable in guiding the design of intervention content and delivery. The second advantage was that patients are often not fully aware of the scientific background of their health conditions [32]. Providing patients with a prototype for a trial and revising the app based on their feedback made our use of resources as effective and efficient as possible. This agrees with the concept of formative research in which health care researchers or practitioners identify a community of interest, describe the features of the community associated with a specific medical issue, and define the initial needs, which are then tested in the population of interest [89]. The qualitative data collected in this stage can provide rich insights into the use of mHealth technology and the most effective engagement strategies [90]. Although we did not deliberately pursue information saturation as part of our qualitative approach, the ideas gathered from different disciplines had many common and overlapping points that guided app development. Focus group interviews with the patients played an additional role. While suggesting several improvements, their attitude toward usefulness and ease of use of the app was positive, indicating the potential value of this app.

Patient feedback affirmed two indicators for measuring the level of acceptance in the technology acceptance model: usefulness and ease of use [91]. This reflects the scientific rigor of our design. The patients also indicated a limitation of the app, that is, insufficient information content and clarity, which is a known factor to affect the success of mHealth systems [32]. This reminded us about the importance of targeting information delivery to fit patients’ health literacy. The main limitation of our prototype was a lack of personalization, which has been identified in previous studies [1,29,30].

Third, most current app-based interventions specifically target bariatric surgery [54,55]. Our research extends the scope of potentially effective mHealth interventions to any elective surgery, making the product much more generalizable to a wider audience.

### Limitations and Future Work

This study had some limitations. First, participants in the focus groups were recruited via purposive sampling, so the diverse demographic groups were not evenly distributed. This could have led to a biased finding of the patient’s level of acceptance and satisfaction with the mHealth app [92]. In the large scope trial, stratified sampling can be used to avoid this problem. Second, the technology is rapidly changing. The current version of the app is relatively simple, despite meeting our identified requirements. Further development is required to develop intelligent and personalized functions. Third, the reuse of lessons may be limited by the small scope of the study at one site. However, the health informatics experts in our team have extensive experience in the development and evaluation of eHealth solutions. Moreover, the app design is underpinned by a carefully selected theory based on sound literature research, leading to robust functionality. Therefore, the design process is useful for other similar learning initiatives.

Future research will be conducted to evaluate the effectiveness of the app, with measures including user satisfaction and perioperative efficiency and outcomes. The app also has the potential to be used postoperatively and preoperatively to provide ongoing motivation and resources to users. This mHealth platform may be particularly useful when face-to-face health care options are limited, such as in regional and remote communities, and during periods with social distancing restrictions. Moreover, the integration of the app-based system with existing electronic health records or tools used by clinicians in the health system could also be further investigated.

### Conclusions

This study reports an innovative co-design approach with clinicians and patients to address the challenges facing participative co-design with patients’ mHealth services that support their self-management of chronic conditions. It presents a detailed process to leverage the experiences of clinicians to produce the initial prototype app. *Hands-on* interaction with the prototype mHealth app in focus group discussions allowed the patients to effectively articulate their needs and expectations for the mHealth app. This research also presents a method to integrate theory into mHealth design, which addresses a missing link in the design of mHealth services that support the patient self-management of chronic conditions. The reported design approach can be generalized to the design of any mHealth services that aim to support the patient self-management of chronic conditions.

## Appendix

Multimedia Appendix 1 Focus group questions.

Multimedia Appendix 2 Comparison of behavioral theories.

Multimedia Appendix 3 Workflow diagram of the prototype.

## Abbreviations

HBM: Health Belief Model
mHealth: mobile health
SCT: Social Cognitive Theory
