# Supplementary material for: A Clinician-Led, Experience-Based Co-Design Approach for Developing mHealth Services to Support the Patient Self-management of Chronic Conditions: Development Study and Design Case
Source: JMIR Mhealth Uhealth. 2021 Jul 20;9(7):e20650. doi: 10.2196/20650 (PMC8335618; doi:10.2196/20650)
Supplement: Multimedia Appendix 1 [file mhealth_v9i7e20650_app1.pdf]

Multimedia Appendix 1 Focus group questions

Introductory

- What phone do you use? iPhone, Android phone or windows phone
- What do you think about this app?

Features/functions

- Are there any features of this app that you would definitely NOT be interested in?
- What features or functions would you like to add to this app?

Push notifications

- How often would you like to receive push notifications from this app?
- At what time of the day do you prefer to receive these notifications? E.g. 12 midday, 4 pm or other time of the day

Expectation

- What goals do you expect to achieve by using this app?
- How do you think this app may help you to achieve these goals?

Concern and benefit

- Would you have any concerns about using this app?
- What benefits do you think this app may bring to you?

Information

- What type of information do you think the app should provide?

Use

- Do you think you will use this app?

App name

- What do you want to name this app?
