# Supplementary material for: A Clinician-Led, Experience-Based Co-Design Approach for Developing mHealth Services to Support the Patient Self-management of Chronic Conditions: Development Study and Design Case
Source: JMIR Mhealth Uhealth. 2021 Jul 20;9(7):e20650. doi: 10.2196/20650 (PMC8335618; doi:10.2196/20650)
Supplement: Multimedia Appendix 2 [file mhealth_v9i7e20650_app2.pdf]

## Multimedia Appendix 2 Comparison of behavioral theories

| Theory | Publishing year | Originator | Main focus | Key factors/procedures | Strengths | Limitations |
|---|---|---|---|---|---|---|
| HBM | 1950s | Social psychologists at the US Public Health Service | People's beliefs about a health problem, perceived benefits, barriers and self-efficacy explain the engagement in health-promoting behavior.<br>HBM is often used to explain/predict individual changes in health behavior. | Perceived susceptibility<br>Perceived severity<br>Perceived benefits<br>Perceived barriers<br>Cues to action<br>Self-efficacy<br>Modifying variables | Providing a theoretical framework for studying cognitive determinants of health-related behaviors<br>Making it easy to implement, apply, and test the models using simplified constructs. | It focuses only on rational reasoning, excluding a person's subconscious or other determinants that dictate his/her acceptance of health behavior. |
| SCT | 1986 | Albert Bandura | Learning occurs in a social context with a dynamic and reciprocal interaction of cognition, environment and behavior.<br>SCT considers the ways that individuals acquire and maintain behavior and the social impact of their behavior. | Reciprocal determinism<br>Behavioral capability<br>Observational learning<br>Reinforcements<br>Expectations<br>Expectancies<br>Self-efficacy<br>Self-control | Concerning important human social behaviors based on long-term accumulation of a large research record. Focusing on important theoretical issues, such as the role of reward in learning, the stability of behavior. | Not a fully systematized, unified theory.<br>Loosely organized, based solely on the dynamic interplay between person, behavior, and environment. It is unclear the extent to which each of these factors into actual behavior and if one is more influential than another. |
| TPB | 1985 | Icek Ajzen | Intention toward attitude, subject norms, and perceived behavioral control, co-shape an individual's behavioral intentions and behaviors. | Normative belief<br>Subjective norm<br>Control beliefs<br>Perceived behavioral control<br>Behavioral intention<br>Behavior | Covering people's non-volitional behavior | Being based on cognitive and rational processing, but ignoring one's actual needs before engaging in a specific action. These needs would affect the behavior regardless of expressed attitudes. |

| Theory | Publishing year | Originator | Main focus | Key factors/procedures | Strengths | Limitations |
|---|---|---|---|---|---|---|
| SDT | 1985 | Edward Deci & Richard Ryan | A person will be motivated to grow and change out of three innate and universal psychological needs.<br>SDT emphasizes the degree to which an individual's behavior is self-determined. | Competence<br>Relatedness<br>Autonomy | Distinguishing between extrinsic and intrinsic motivation. | Being confined to explaining behavioral motivations exclusively in traditional settings and thereby neglecting other possibilities, which may lead to the reproduction of prejudice and inequality. |
| TTM | 1977 | James O. Prochaska & Carlo Di Clemente | Individuals move through six periodic stages of change to habitual behavior. | Precontemplation<br>Contemplation<br>Preparation<br>Action<br>Maintenance<br>Termination/Relapse | Providing a framework for categorizing individuals' readiness to change their behavior. | Uneasy to implement as the boundaries between various stages are blurred<br>Applying to only a single behavior and ignoring the impact of other social contexts and behaviors. |
| COM | 1999 | Bracht et al | Encouraging participatory decision-making processes to enable communities to identify fundamental health problems and corresponding solving strategies actively. | Community analysis<br>Design initiation<br>Implementation<br>Maintenance-consolidation<br>Dissemination-reassessment | There are strong feasibility and influence, through community health workers collectively mobilized to develop plans to achieve health goals. | Lacking specific explanation and corresponding measures for individual or relationship level problems in the Health Ecological Model. |
| DOI | 1962 | Everett Rogers | Explaining how, why, and at what rate new ideas/ technologies develop and spread over time. | Relative Advantage<br>Compatibility<br>Complexity<br>Trialability<br>Observability | Accelerating the adoption of many public health programs which are designed to changing the behavior of social systems. | Not promoting a participatory approach to adopt a public health program.<br>Focusing on community-level in the Health Ecological Model. |

HBM: Health Belief Model; SCT: Social Cognitive Theory; TPB: Theory of Planned Behavior; SDT: Self-determination Theory; TTM: Transtheoretical Model; COM: Community Organization Model; DOI: Diffusion of Innovation Theory
