# Supplementary figures and images for: A Clinician-Led, Experience-Based Co-Design Approach for Developing mHealth Services to Support the Patient Self-management of Chronic Conditions: Development Study and Design Case
Source: JMIR Mhealth Uhealth. 2021 Jul 20;9(7):e20650. doi: 10.2196/20650 (PMC8335618; doi:10.2196/20650)

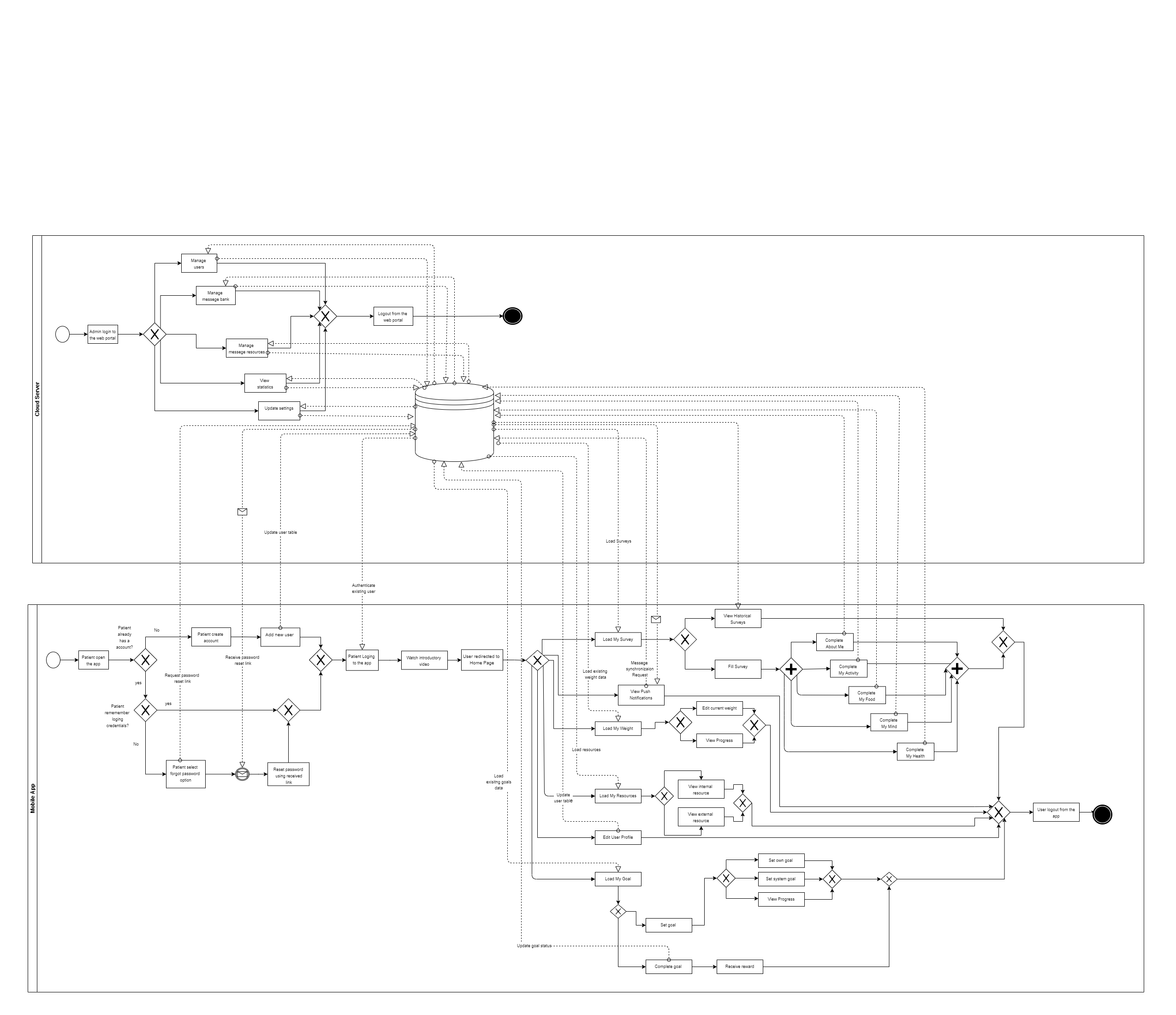

Supplement: Multimedia Appendix 3 [file mhealth_v9i7e20650_app3.png]
